# Effect and Regulation of the NLRP3 Inflammasome During Renal Fibrosis

**DOI:** 10.3389/fcell.2019.00379

**Published:** 2020-01-24

**Authors:** Hong Zhang, Zhengchao Wang

**Affiliations:** Provincial Key Laboratory for Developmental Biology and Neurosciences, Key Laboratory of Optoelectronic Science and Technology for Medicine of Ministry of Education, College of Life Sciences, Fujian Normal University, Fuzhou, China

**Keywords:** NLRP3 inflammasome, inflammatory response, pyroptosis, mitochondrial regulation, myofibroblast differentiation, renal fibrosis

## Abstract

Renal fibrosis is a common pathological process where certain primary or secondary kidney diseases can continue to progress to the end-stage of the kidney disease; however, the molecular mechanisms underlying renal fibrosis remain unclear. Recently, research focusing on examining the function of inflammasomes has attracted a great deal of attention, and data derived from these research projects have increased our understanding of the effects and regulation of inflammasomes during renal fibrosis. Based on this, the present review summarizes recent findings in regard to NLRP3 inflammasome functions during various kidney diseases, and these findings indicate that the NLRP3 inflammasome not only mediates the inflammatory response but is also associated with pyroptosis, mitochondrial regulation, and myofibroblast differentiation during renal fibrosis. These novel findings provide us with a more in-depth understanding of the pathogenesis of renal fibrosis and will aid in the identification of new targets that can be used for the prevention and treatment of this disease.

## Introduction

Renal fibrosis is one of the main underlying causes of end-stage kidney disease (Liu, 2006). Inflammasomes are intracellular multiprotein complexes that can trigger the host defense response (Mulay, 2019). The NLRP3 inflammasome is currently the most studied and characterized inflammasome and acts as an important danger-recognition platform to protect the body from pathogenic microbial molecules and endogenous risk factors (Medzhitov, 2008). Additionally, the NLRP3 inflammasome mediates the maturation and release of proinflammatory cytokines to initiate excessive inflammatory reactions, which cause irreversible damage to the body (Kanneganti et al., 2007). A large number of studies have also shown that the NLRP3 inflammasome is involved in the development of chronic kidney diseases (CKD) (Wu et al., 2018; Li et al., 2019; Mulay, 2019). Therefore, the role and regulation of the NLRP3 inflammasome during renal fibrosis are here reviewed.

## Renal Fibrosis

Based on specific molecular and cellular mechanisms, the process of renal fibrosis can be artificially divided into four overlapping stages, namely priming, activation, execution, and progression (Liu, 2011). Unresolved inflammation after a sustained injury facilitates the fibrogenic stage (the priming stage), where immune cells infiltrate into the kidney and secrete various factors, including chemokines, cytokines, and reactive oxygen species (ROS), that ultimately result in further renal injury (Imig and Ryan, 2013). During the activation stage, the cells undergo trans-differentiation and increase their expression of alpha smooth muscle actin (α-SMA) and their secretion of extracellular matrix (ECM) (Chevalier et al., 2009). During the execution stage, ECM components are synthesized and deposited within the interstitial space and modified to resist proteolytic enzymes (Liu, 2006). Progression represents the final stage of fibrosis, and this stage involves several types of renal injuries, including tubular injury, atrophy, and chronic hypoxia (Hodgkins and Schnaper, 2012; Pan et al., 2013).

Renal fibrosis is the final outcome of various CKDs, including diabetic nephropathy (DN), chronic glomerulonephritis, crystal-related nephropathy, IgA nephropathy, and others (Liu, 2006). It is characterized by a number of events such as the abnormal accumulation of ECM, a decrease in or atrophy of renal tubules and intact renal units, and a decrease in glomerular filtration rate, all of which can lead to irreversible damage to renal functions (Nogueira et al., 2017). It is believed that transforming growth factor (TGF)-β/Smad signaling plays an important role in renal fibrosis, where this signaling not only regulates the production and degradation of ECM but also participates in the epithelial-to-mesenchymal transition (EMT) to form myofibroblasts (Zhang K. et al., 2013). Connective tissue growth factor (CTGF), a downstream molecule of TGF-β/Smad, is the key factor for tissue fibrosis, acting on the kidney in an autocrine/paracrine manner to promote the abnormal deposition of ECM and fibrosis (Mansour et al., 2017). Recent findings, however, indicate that NLRP3 and other inflammasomes are also involved in renal fibrosis (Wu et al., 2018; Mulay, 2019).

## The NLRP3 Inflammasome

Inflammasomes generally consist of a pattern recognition receptor (PRR), an apoptosis-associated speck-like protein (ASC), and the cysteine protease caspase-1 (Kanneganti et al., 2007). To date, five families of PRRs have been described, and these include Toll-like receptors (TLRs), nucleotide-binding oligomerization domain (NOD)-like receptors (NLRs), absent in melanoma 2 (AIM2)-like receptors (ALRs), Rig-I-like receptors (RLRs), and C-type lectin receptors (CLRs) (Wang and Yi, 2015; Komada and Muruve, 2019). Existing evidence suggests that some members of the NLR family and ALR family, including NLRP1, NLRP3, NLRC4, AIM2, and pyrin, can form inflammasomes (Martinon et al., 2002; Fernandes- Alnemri et al., 2009; Hornung et al., 2009; Broz et al., 2010; Xu et al., 2014). Among these, NLRP3 is the most studied inflammasome.

### Composition of the NLRP3 Inflammasome

The NLRP3 protein contains three different domains, namely a central nucleotide-binding NACHT domain (NOD domain), C-terminal leucine-rich repeat (LRR), and an N-terminal pyrin domain (PYD). The NOD domain is mainly responsible for self-oligomerization during activation, the C-terminus is considered to be the recognition domain for different ligands, and the N-terminus primarily mediates the interaction with proteins (Strowig et al., 2012). As an adapter protein, ASC possesses a PYD that interacts with the NLRP3 protein and a caspase recruitment domain (CARD) that interacts with pro-caspase-1, ultimately forming the NLRP3 inflammasome (Medzhitov, 2008).

### Activation of the NLRP3 Inflammasome

It is well established that a variety of exogenous stimuli such as bacteria, viruses, silica crystals, and ultraviolet rays can promote the activation of the NLRP3 inflammasome (Martinon et al., 2002; Dostert et al., 2008; Allen et al., 2009; Wu et al., 2010). Additionally, endogenous stimuli, including ATP, uric acid crystal salts, and active oxygen, can also activate it (Mariathasan et al., 2006; Martinon et al., 2006; Zhou et al., 2010). Although the specific mechanism remains unclear, it is generally accepted that potassium (K^+^) efflux (Munoz-Planillo et al., 2013), the generation of ROS (Lawlor and Vince, 2014), and lysosomal damage that coincides with the release of endogenous cathepsins into the cytosol (Hornung et al., 2008) influence the activation of the NLRP3 inflammasome.

Two canonical steps occur during the activation of the NLRP3 inflammasome. First, microbial molecules or endogenous factors promote the expression of NLRP3, pro-IL-1β, and pro-IL-18 through the NF-κB pathway (Bauernfeind et al., 2009). Second, these stimuli induce the oligomerization and activation of NLRP3 and the recruitment of the adapter protein ASC and pro-caspase-1, the latter of which undergoes autoproteolytic cleavage into caspase-1 to activate pro-IL-1β and pro-IL-18 to produce active cytokines (Martinon et al., 2002; Lamkanfi and Dixit, 2014; Man and Kanneganti, 2015).

An alternative activation pathway for NLRP3 inflammasomes involves human caspase-4, caspase-5, and murine caspase-11 and their ability to directly recognize lipopolysaccharides and toxins, to create macromolecules, and to cleave gasdermin D (GSDMD) to release its N-terminus, ultimately resulting in the formation of membrane pores. Caspase-11 can also cleave the pannexin 1 channel protein, resulting in ATP leakage, efflux of K^+^, influx of Ca^2+^, and, finally, NLRP3 inflammasome activation and pyroptosis (Yang et al., 2005; Broz and Monack, 2013; Shi et al., 2017).

## NLRP3 Inflammasome and Renal Fibrosis

Increasing evidence strongly indicates that the expression levels of NLRP3 and caspase-1 are significantly elevated in the kidneys of CKD or fibrosis patients (Vilaysane et al., 2010; Ke et al., 2018), suggesting that the NLRP3 inflammasome may be activated and involved in the regulation of renal fibrosis.

### NLRP3 Inflammasome and Inflammation During Renal Fibrosis

Classical immune cells such as resident dendritic cells and infiltrating macrophages can express all of the NLRP3 components and can cause cell death owing to the activation of caspase-1 in the kidney (Franke et al., 2012). It has been previously reported that renal tubular epithelial cells can express and release IL-18, indicating that the NLRP3 inflammasome and caspase-1 are also present within renal tubular epithelial cells (Faust et al., 2002; Franke et al., 2012). Zhang et al. (2012) demonstrated that NLRP3 and ASC are expressed in glomerular podocytes, suggesting that podocytes can also form inflammasomes. Studies examining unilateral ureteral obstruction (UUO) mouse models and 5/6-nephrectomized mice revealed that these mice had increased renal matrix accumulation, elevated levels of phosphorylated NF-κB, and activated NLRP3 inflammasomes (Seo et al., 2019; Wen et al., 2019). Artemisinin may down-regulate the NF-κB/NLRP3 signaling pathway, mitigating renal tubulointerstitial inflammation and fibrosis in 5/6-nephrectomized rats (Wen et al., 2019). Guo et al. (2016) reported that the use of anti-IL-1β monoclonal antibodies to neutralize the expression of IL-1β within the lungs and serum can reduce silica-induced inflammatory responses in the heart and kidney and attenuate renal fibrosis in mice.

Some researchers have found that oxalate crystals activate NLRP3 to promote IL-1β release and macrophage infiltration, events that are critical in the early stages of crystal-induced renal fibrogenesis (Ermer et al., 2016). The Shen Shuai II Recipe, which has been used clinically for >20 years and has been confirmed to be effective for improving renal function and fibrosis, can effectively inhibit the activation of the NLRP3/ASC/Caspase-1/IL-1β axis and reduce inflammatory infiltration (Meng et al., 2019). Immunoglobulin A nephropathy (IgAN) characterized by glomerular proliferation and renal inflammation is the most common form of glomerulonephritis. IgA deposition, mesangial matrix expansion, and glomerular fibrosis were found to be significantly increased in IgAN rats. It has been reported that icariin, a flavonoid derived from the Chinese herb epimedium, which possesses anti-inflammatory effects, can dramatically block the nuclear transport of NF-κB, inhibit the activation of the NLRP3 inflammasome, and reduce the production of downstream pro-inflammatory cytokines to ameliorate IgAN (Zhang et al., 2017). Recently, DN has become the second leading cause of end-stage renal disease, while glomerulonephritis remains the leading cause. Immunohistochemical results have revealed positive staining for thioredoxin-interacting protein (TXNIP), NLRP3, and IL-1β within kidney tissues obtained from diabetic rats (Feng et al., 2016). NLRP3, caspase-1, IL-1β, and IL-18 expressions were increased markedly in mesangial cells and renal tubular epithelial cells that were treated with high amounts of glucose *in vitro* (Feng et al., 2016). High glucose and uric acid levels can mediate inflammatory responses within tissues via the ROS/TXNIP/NLRP3/IL-1β/IL-18 axis. Dihydroquercetin, an important natural dihydroflavone, exerts renal protection effects during DN by suppressing ROS and the NLRP3 inflammasome (Ding et al., 2018). Recently, Yaribeygi et al. (2019) collected and summarized a large number of studies and found that certain antidiabetic drugs such as SGLT2 inhibitors (Ye et al., 2017; Birnbaum et al., 2018), biguanides (Woo et al., 2014; Li A.Y. et al., 2016), thiazolidinediones (Wang Y. et al., 2017), and DPP-4 inhibitors (Birnbaum et al., 2016) can also modulate NLRP3 inflammasome activity to prevent the development of DN. Together, these studies have consistently found that the development of various kidney diseases such as crystal-related nephropathy, IgA nephropathy, and DN are associated with the activation of the NLRP3 inflammasome, which mediates inflammatory responses through the NLRP3/CAS1/IL-1β/IL-18 axis and participates in the early stage of renal fibrosis. Most studies, however, focused on the role of traditional Chinese herbal medicines or their potent components for the treatment of renal diseases, and their results have consistently demonstrated that the inhibition of the NLRP3 inflammasome is related to the protective effect of these compounds on the kidney.

Additionally, several studies have suggested that certain cytokines can also regulate the activation of the NLRP3 inflammasome to mediate renal inflammation and fibrosis (Chi et al., 2017; Wang S.F. et al., 2017). For example, Chi et al. (2017) reported that recombinant IL-36a contributes to the activation of the NLRP3 inflammasome in renal tubular epithelial cells, macrophages, and dendritic cells during renal inflammation and fibrosis. IL-22 can down-regulate the NLRP3/caspase-1/IL-1β pathway and decrease the expression of fibronectin and type IV collagen in renal mesangial cells induced by high glucose, suggesting that IL-22 plays an anti-inflammatory and anti-fibrosis role by inhibiting the activation of the NLRP3 inflammasome (Wang S.F. et al., 2017).

### NLRP3 Inflammasome and Pyroptosis During Renal Fibrosis

Pyroptosis is a recently discovered pro-inflammatory programmed death pattern that is divided into a classical caspase-1-dependent pyroptosis and a non-caspase-1-dependent pyroptosis. The non-caspase-1-dependent pyroptosis is mediated by human caspase-4, caspase-5, and murine caspase-11 (Baker et al., 2015); however, the morphological changes associated with the two pathways are similar. NLRP3, caspase-1, IL-18, and IL-1β are key factors that are required for caspase-1-dependent pyroptosis. Caspase-1-dependent pyroptosis involves four major steps, namely inflammasome assembly, the activation of pro-caspase-1, the maturation of inflammatory factors (IL-1β and IL-18), and the cleavage of GSDMD (Miao et al., 2010). Pyroptosis is implicated in the development of various kidney diseases, such as ischemia–reperfusion acute kidney injury, DN, crystal-related nephropathy, and renal fibrosis (Yang et al., 2014; Hutton et al., 2016; Li X. et al., 2016; Guo et al., 2017). Crystals deposited within the renal tubules activate the NLRP3 inflammasome and downstream-related signaling molecules, ultimately causing renal tubular epithelial pyroptosis (Hutton et al., 2016). A recent study also found that caspase-11 stimulates the maturation of IL-1 to promote renal fibrosis by activating caspase-1 (Miao et al., 2019). It can be observed that pyroptosis also participates in the progression of renal fibrosis. Further studies focusing on the interaction between pyroptosis and renal diseases may aid in the development of strategies that can be used to slow the progression of renal fibrosis.

### NLRP3 Inflammasome and Mitochondrial Regulation During Renal Fibrosis

Almost all NLRP3 activators can induce ROS generation (Zhou et al., 2010). Zhou et al. (2010) reported that ROS can dissociate TXNIP from thioredoxin, after which TXNIP binds to NLRP3 to activate the NLRP3 inflammasome. Kim et al. (2018) suggested that hypoxia can induce a significant increase in NLRP3 that is independent of ASC, caspase-1, and IL-1β. NLRP3 in renal tubular cells is re-localized from the cytosol to the mitochondria during hypoxia where it binds to mitochondrial antiviral signal protein (MAVS). The deletion of NLRP3 or MAVS in renal tubular cells attenuates mitochondrial ROS production and the depolarization of the mitochondrial membrane potential under hypoxia (Kim et al., 2018). NLRP3 deletion mutants exhibit normal mitochondrial morphology and DNA copy numbers in renal tubule cells, suggesting that NLRP3 deficiency can improve mitochondrial abnormality (Gong et al., 2016). Guo et al. (2017) have demonstrated that NLRP3 deletion can reverse the morphological and functional damage to mitochondria caused by unilateral ureter obstruction and improve CKD symptoms to alleviate renal fibrosis.

It should be noted that autophagosomes can negatively regulate the activation of the NLRP3 inflammasome (Saitoh et al., 2008). This mechanism may be related to a decrease of ASC, the phosphorylation of the NLRP3 protein, and the purge of mitochondrial ROS (Spalinger et al., 2016, 2017; Ko et al., 2017; Nurmi et al., 2017). Certain studies have demonstrated that autophagy induced by TGF-β can mitigate the progression of tubular epithelial cells fibrosis in obstructive renal tissue (Ding et al., 2014; Lim et al., 2019). In DN, mitochondrial autophagy is related to the regulation of M1/M2 macrophages, reduced inflammation, and further damage (Zhao et al., 2017). Hu et al. (2018) found that autophagy was increased and the degradation of NLRP3 was accelerated after treatment with Weicao Capsules in rats with uric acid nephropathy and that this contributed to the alleviation of inflammation, renal tissue crystallization, and renal interstitial fibrosis. Based on these findings, we speculate that a complex interaction exists among ROS, the NLRP3 inflammasome, and autophagy during the progress of fibrosis.

### NLRP3 Inflammasome and Myofibroblast Differentiation During Renal Fibrosis

**FIGURE 1:**
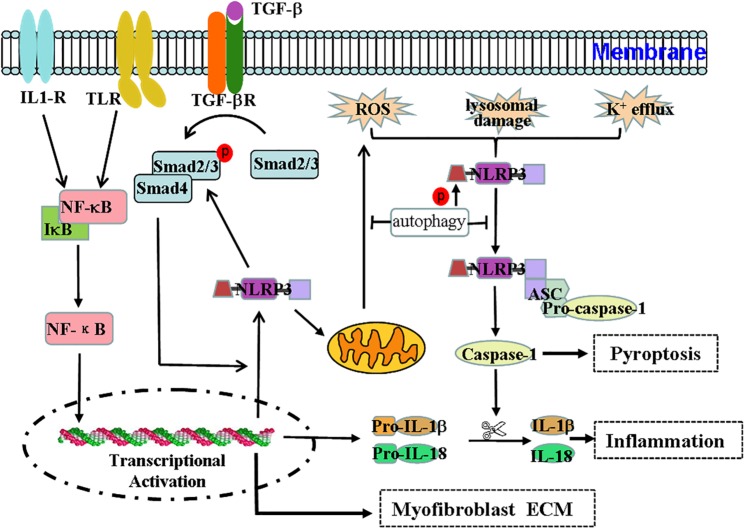
Effect and regulation of the NLRP3 inflammasome during renal fibrosis. The NLRP3 inflammasome not only mediates the inflammatory response but is also associated with pyroptosis, mitochondrial regulation, and myofibroblast differentiation during renal fibrosis. Regulation of the NLRP3 inflammasome during renal fibrosis is mediated by the following pathways. (1) Microbial molecules or endogenous risk factors up-regulate the expression of NLRP3, pro-IL-1β, and pro-IL-18 through the NF-κB signaling pathway to induce events such as K^+^ efflux, the generation of ROS or lysosomal damage, and others. (2) This triggers the oligomerization and activation of NLRP3 and the recruitment of the adapter protein ASC and pro-caspase-1. Pro-caspase1 undergoes autoproteolytic cleavage into bioactive caspase-1, which in turn acts on pro-IL-1β and pro-IL-18 to produce IL-1β and IL-18 to mediate inflammation and pyroptosis. (3) TGF-β signaling participates in the transdifferentiation of myofibroblasts and the production of ECM. (4) The TGF-β/Smad pathway can stimulate the expression of NLRP3, which is also involved in TGF-β-mediated Smad2/3 phosphorylation. Thus, the interaction between the NF-κB/NLRP3/caspase-1/IL-1β/IL-18 axis and the TGF-β/Smad signaling pathway may form a pathogenic cycle that leads to the development of renal fibrosis. (5) Additionally, NLRP3 may damage mitochondrial morphology and induce mitochondrial ROS production, ultimately promoting NLRP3 inflammasome activation. (6) Autophagy can negatively regulate the NLRP3 inflammasome by decreasing the levels of ASC and the phosphorylation of the NLRP3 protein and by purging mitochondrial ROS.

*In vitro* experiments have demonstrated that IL-1β can induce the development of CKD and the transformation of renal tubular epithelial cells into myofibroblasts (Zhang et al., 2003). NLRP3 appears to exert non-classical effects on interstitial fibrosis of renal tubular epithelial cells, such as regulating NLRP3 in TGF-β signaling in renal tubular epithelial cells (Wang et al., 2013). Previous research has demonstrated that hyperuric acid increases the expression of NLRP3/ASC and activates the inflammation-associated caspase-1 and inflammation-independent smad2/3 pathways (Romero et al., 2017). The ultrastructural colocalization of NLRP3 and smad2/3 indicates a physical interaction between these two molecules (Romero et al., 2017). NLRP3 expression increases after TGF-β stimulation in renal tubular epithelial cells, and the expression of NLRP3 is associated with epithelial-to-mesenchymal transition, α-SMA expression, and matrix metalloproteinase (MMP9) expression (Wang et al., 2013). Compared to levels in wild-type diabetic mice, the expression of TGF-β and CTGF and the phosphorylation of Smad2/3 are dramatically reduced in NLRP3^–/–^ diabetic mice in a manner that can ameliorate renal function (Wu et al., 2018). Additionally, the overexpression of NLRP3 in 293T cells leads to increased phosphorylation and activity of Smad3 (Wang et al., 2013). Thus, not only does TGF-β rely on Smad3 to increase NLRP3 expression, but NLRP3 is also involved in the expression of TGF-β and TGF-β-mediated Smad2/3 phosphorylation. Autophagy in distal tubular epithelial cells exerts a protective effect on tubulointerstitial fibrosis after UUO via modulating the expression of TGF-β and IL-1β (Nam et al., 2010). Taken together, these findings indicate that autophagy, NLRP3, and TGF-β exhibit a complex functional relationship (Figure 1).

## Other Inflammasomes and Renal Fibrosis

In addition to the NLRP3 inflammasome, other inflammasomes such as NLRP1, NLRC4, and AIM2 are also related to some types of CKD.

The NLRP1 inflammasome was initially discovered to respond to the *Bacillus anthracis* lethal factor. A recent study demonstrated that two NLRP1 gene variants, rs11651270 and rs2670660, are associated with a decreased risk of development of DN, suggesting that NLRP1 may play a critical role in the etiology of DN (Soares et al., 2018). The molecular mechanisms underlying the participation of NLRP1 in CKD, however, remain unclear.

Furman et al. (2017) reported that, compared to the levels in a young group, the expression of NLRP3 and NLRC4 was significantly increased in aging kidney tissue. Age-associated renal diseases are related to the NLRC4 inflammasome (Furman et al., 2017). NLRC4 deficiency resulted in diminished disease progression in diabetic mice (Yuan et al., 2016).

Unlike the other three inflammasomes, AIM2 is composed of a DNA-sensing hematopoietic interferon-inducible nuclear protein that is comprised of 200 amino acids (HIN200) and a PYD domain (Man et al., 2015; Hu et al., 2016). Therefore, AIM2 can form a DNA-sensing inflammasome in combination with ASC and caspase-1 that mediates inflammation and pyroptosis (Gray et al., 2016). AIM2 deficiency attenuates renal injury, inflammation, and fibrosis in mouse UUO models (Komada et al., 2018). Additionally, recent findings suggest that AIM2 expression is increased in renal tubular epithelial cells and infiltrating leukocytes derived from patients with DN or hypertensive nephrosclerosis (Komada et al., 2018). Findings by Zhang W.J. et al. (2013) also indicate that AIM2 expression is increased in lupus patients and is closely correlated with the severity of disease in systemic lupus erythematosus patients. AIM2 may also be involved in the pathogenesis of lupus nephritis (Kimkong et al., 2009; Zhang W.J. et al. (2013)).

## Conclusion

The NLRP3 inflammasome is a complex platform that is responsible for the activation of cytosolic polyprotein caspase, which is involved in the maturation and release of IL-1β and IL-18, which systemically establishes an inflammatory environment within the kidney under appropriate stimuli. The typical NLRP3/ASC/caspase-1/IL-1β/IL-18 axis promotes the pathophysiology of various kidney diseases by mediating inflammation, and this is likely a critical priming mechanism for renal fibrosis. Additionally, the NLRP3 inflammasome is associated with pyroptosis, a process that is also involved in renal fibrosis. The inflammation-independent NLRP3 inflammasome is also closely associated with mitochondrial regulation and TGF-β/Smad signaling. The existence of crosstalk between NLRP3 and the TGF-β/Smad signaling pathway indicates that NLRP3 plays an important role in myofibroblast differentiation and ECM accumulation. Granata et al. (2015) reported that the NLRP3 inflammasome is activated and the production of mitochondrial ROS is elevated in immunocompetent peripheral cell lines isolated from uremic patients undergoing dialysis treatment. Therefore, the NLRP3 inflammasome pathway may serve as a valuable prophylactic and therapeutic target for the treatment of renal fibrosis and may also provide a potential target for minimizing the severe clinical complications observed in CKD patients with advanced renal impairment.

## Author Contributions

HZ wrote the manuscript. ZW revised the manuscript. Both authors read and approved the final version of the manuscript for publication.

## Conflict of Interest

The authors declare that the research was conducted in the absence of any commercial or financial relationships that could be construed as a potential conflict of interest.
